# Demographic characteristics and disease severity associated with IgA/IgG deposition patterns in autoimmune bullous diseases: a cohort study based on a registry database

**DOI:** 10.3389/fimmu.2025.1565073

**Published:** 2025-05-26

**Authors:** Jishu Li, Xun Feng, Mi Wang, Hongjie Liu, Mei Yang, Jiyun Pang, Min Zou, Yue Xiao, Xiwen Zhang, Hongxiang Hu, Yuxi Zhou, Yazan Moufaq Alqusseireen, Wei Yan, Xingli Zhou, Wei Li

**Affiliations:** ^1^ Department of Dermatology and Venereology and Rare Diseases Center, West China Hospital, Sichuan University, Chengdu, China; ^2^ Institutes for Systems Genetics, Frontiers Science Center for Disease-related Molecular Network, West China Hospital, Sichuan University, Chengdu, China; ^3^ Department of Pathology, West China Hospital, Sichuan University, Chengdu, China

**Keywords:** autoimmune bullous disease, direct immunofluorescence, IgA, pemphigus, pemphigoid

## Abstract

**Background:**

Direct immunofluorescence (DIF) microscopy is the gold standard for diagnosing autoimmune bullous diseases (AIBDs), but the clinical significance of IgA and IgG co-deposition was unclear.

**Objective:**

Investigate the demographic differences and disease severity among different IgG/IgA deposition patterns in DIF.

**Methods:**

We conducted a retrospective cohort study based on a registry database that analyzed demographic data, involvement sites, and immunofluorescence patterns of patients with DIF biopsy. Patients were categorized into intercellular (group A) and basement membrane zone (group B) deposition patterns. Logistic regression models assessed associations between deposition status and demographic characteristics. Disease severity and prognosis were analyzed retrospectively through subgroup analyses.

**Results:**

In group A, female gender (OR = 1.665, *P* = 0.011) and stronger IgG deposition (OR = 3.881, *P* < 0.001) were associated with IgA and IgG co-deposition. In group B, female gender (OR = 1.382, *P* = 0.002), stronger IgG deposition (OR = 2.673, *P* < 0.001), and mucosa tissue (OR = 3.052, *P* < 0.001) were associated with IgA and IgG co-deposition. IgA and IgG co-deposition in group A was associated with higher Pemphigus Disease Area Index scores (*P* = 0.036), while in group B, it correlated with mucosal involvement (*P* = 0.007). No differences in the proportion of disease severity scores improvement after 6 months of standard treatment were found in both groups.

**Conclusions:**

Female gender, stronger IgG deposition, and mucosa tissue are key factors affecting IgA and IgG co-deposition in AIBD patients. For clinical correlation, patients with IgA and IgG co-deposition in pemphigus exhibit more severe disease severity compared with those with IgG deposition only, while patients with co-deposition in pemphigoid are more prone to mucosal involvement.

## Introduction

Autoimmune bullous diseases (AIBDs) are a group of rare, chronic, and potentially fatal autoimmune diseases characterized by autoantibodies against structural proteins in the epidermis or basement membrane of the skin and mucosa (1, 2). The clinical features of AIBD patients are vesicles, blisters, pustules, erosions, excoriations, and erythema on the skin and mucous membranes (3). Direct immunofluorescence (DIF) microscopy showing immunoglobulin and/or complement component 3 (C3) deposition remains the gold standard for diagnosing AIBDs (4). When a patient is suspected of AIBD, a biopsy from the skin or mucosa is recommended for histopathological examination and DIF.

**Figure 1:**
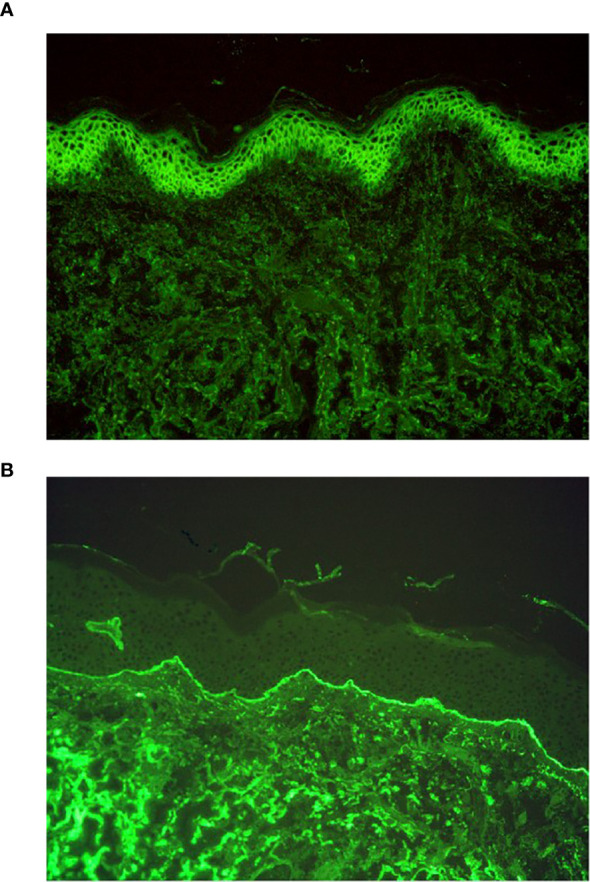
DIF results of IgA pemphigus and linear IgA bullous dermatosis. **(A)** DIF result of IgA pemphigus, IgA net-like deposition intercellularly (original magnification, ×200). **(B)** DIF result of linear IgA bullous dermatosis, IgA line-like deposition along the BMZ (original magnification, ×200).

In AIBDs, immunoglobulin G (IgG) and C3 can deposit intercellularly and/or in the basement membrane zone (BMZ) through DIF, typically net-like or line-like (5). In addition to IgG and C3, DIF may also reveal the presence of immunoglobulin A (IgA), immunoglobulin M (IgM), and immunoglobulin E (IgE) in certain cases (6–8). Specifically, patients demonstrating intercellular IgA (**Figure 1A**) deposition or localized BMZ IgA (**Figure 1B**) are often diagnosed with IgA pemphigus or linear IgA bullous dermatosis (LABD) (9, 10). Notably, IgA can co-deposit with IgG, and some studies have categorized this subset as a special type of pemphigus or pemphigoid (11–13). However, the clinical implications of the co-deposition pattern remain unclear, which suggests that their intricate interplay necessitates further systematic investigation.

In this study, we aimed to observe the deposition patterns of immunoglobulin in AIBDs and explore the factors associated with IgA and IgG co-deposition. Moreover, we compared the clinical differences between patients with IgA and IgG co-deposition and IgG deposition only by reviewing their disease severity, involvement sites, and the proportion of disease severity scores improvement after 6 months of standard treatment. We hope this study can reveal the demographic characteristics of patients with IgA and IgG co-deposition and provide new insights into the diagnosis of these patients.

## Methods

This was a retrospective cohort study based on the Autoimmune Bullous Disease Cohort of West China Hospital (AIBD-WCH). The AIBD-WCH was established in 2017 and includes more than 1,000 confirmed pemphigus and pemphigoid patients from October 2007, which was approved by the biomedical research ethics committee of West China Hospital of Sichuan University (approval number: 2017-241). We collected the demographic data (age and sex), involvement sites (mucosal and/or cutaneous involvement), and immunofluorescence patterns (type and fluorescence intensity) of patients in the AIBD-WCH from October 2007 to December 2023. The patients needed to meet the following inclusion criteria (1): the biopsy tissue was skin or mucosa, (2) with immunoglobulins or C3 deposition intercellularly or in the BMZ. Exclusion criteria: the patients primarily diagnosed with lupus erythematosus, vasculitis, or other diseases outside of AIBDs. All patients were allocated into two groups based on deposition pattern: group A (depositing intercellularly) and group B (depositing in the BMZ). In each group, the patients were sorted by deposition of IgA or IgG. The fluorescence intensity was determined by two pathologists using a semi-quantitative scoring method, ranging from 1+ (weakest) to 4+ (strongest).

Based on the collected cohort, we performed clinical correlation analyses in patients with complete clinical information. For group A (pemphigus), PDAI scores were recorded and desmoglein 1 (Dsg1) and desmoglein 3 (Dsg3) autoantibody titers were retrieved. For group B (pemphigoid), BPDAI scores were collected. Involvement sites were recorded in both groups. Disease severity was classified according to guideline-recommended cutoffs: group A (PDAI): ≤15 as mild, >15 as moderate-to-severe; group B (BPDAI): ≤19 as mild, >19 as moderate-to-severe (14, 15). For patients who received treatment in line with guideline recommendations and had follow-up data beyond 6 months, we collected PDAI or BPDAI scores at the 6-month mark. We conducted subgroup analyses to compare disease severity, mucosal involvement, and improvement in disease severity scores after 6 months of treatment between patients with IgA/IgG co-deposition and those with IgG deposition only.

The statistical analysis was completed through SPSS Statistics version 26.0. We described the demographical characteristics and immunofluorescence patterns among different deposition statuses. The categorical variables were presented as count (percentage), and continuous variables as mean (standard deviation, SD) or median (IQR). The differences in the variables among multiple subgroups were managed using Pearson’ χ2 test (or Fisher’s exact test) or a one-way analysis of variance test. Associations between variables and deposition patterns were analyzed using the chi-square test, Mann–Whitney test, Kruskal–Wallis test, and logistic regression. A *p*-value < 0.05 was considered significant.

## Results

### Study population

**Figure 2:**
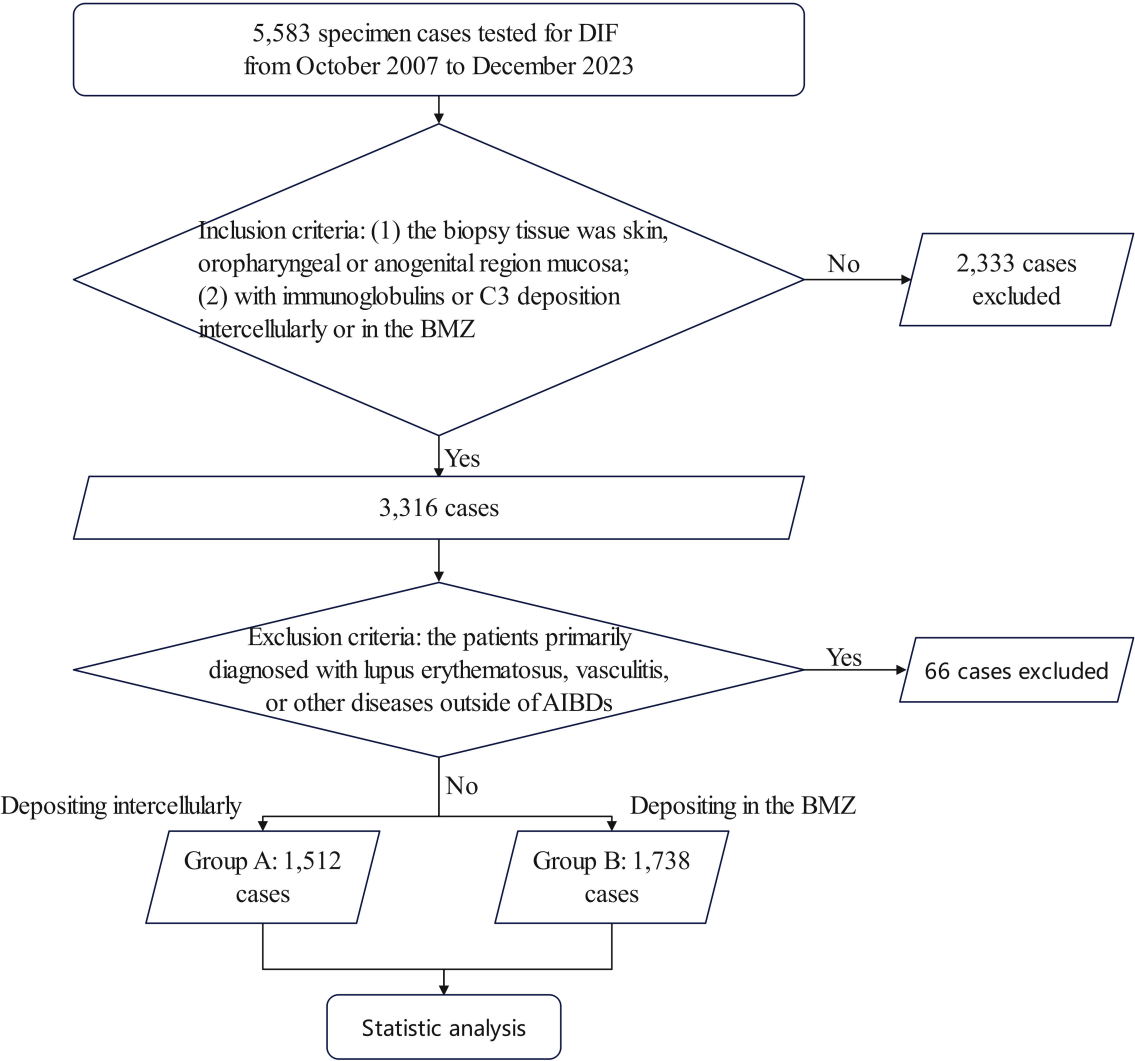
Flow-diagram chart of the study. Inclusion criteria: (1) the biopsy tissue was skin, oropharyngeal or anogenital region mucosa; (2) with immunoglobins or C3 deposition intercellularly or in the BMZ. Exclusion criteria: the patients with a primary diagnosis of lupus erythematosus. Group A: depositing intercellularly; Group B: depositing in the BMZ; BMZ, basement membrane zone; LE, lupus erythematosus.

A total of 5,583 specimen cases were tested for DIF, and finally, 3,250 cases were included (**Figure 2**). Among them, 1,512 (46.52%) showed antibodies, including IgA, IgG, and IgM, net-like depositions, while 1,738 (53.48%) showed line-like deposition of antibodies. In the analysis of disease severity and tissue involvement, we ultimately collected clinical information from 544 patients in group A (59 with IgA and IgG co-deposition and 485 with IgG deposition only) and 194 patients in group B (46 with IgA and IgG co-deposition and 148 with IgG deposition only). In the study of changes in disease activity, we collected data from 306 patients in group A (39 with IgA and IgG co-deposition and 267 with IgG deposition only) and 81 patients in group B (17 with IgA and IgG co-deposition and 64 with IgG deposition only).

### The demographic characteristics and immunofluorescence patterns of AIBD patients

In group A, there were 1,512 cases, predominantly females (874 cases, 57.80%), with a median age of 50 years old. The number of skin specimens (792 cases, 52.38%) was comparable to mucosal specimens (720 cases, 47.62%). Regarding deposition patterns, 1,310 cases (86.64%) exhibited IgG deposition only, nine cases (0.59%) exhibited IgA deposition only, 128 cases (8.47%) showed both IgA and IgG deposition, and nine cases (0.59%) displayed IgA, IgG, and IgM depositing together. None had IgA and IgM co-deposition.

In group B, there were 1,738 cases, with a higher proportion of females (974 cases, 56.04%) and a median age of 59 years old. Most tissues submitted for examination were skin specimens (1,084 cases, 62.37%). In terms of deposition, 792 cases (45.57%) had only IgG deposited, 50 cases (2.88%) had IgA deposition only, 295 cases (16.97%) had both IgA and IgG deposited, and 96 cases (5.52%) had IgA, IgG, and IgM depositing together. Only six cases (0.34%) had IgA and IgM co-deposition. The demographic characteristics and immunofluorescence patterns of AIBD patients with biopsy are shown in **Supplementary Table S1**.

### Factors associated with IgA and IgG co-deposition

In this study, IgA and IgG co-deposition was the most common deposition pattern of IgA. The univariable analysis results are presented in **Table 1**. In group A, the OR for IgA and IgG co-deposition associated with female gender was 1.665 (95% CI, 1.131 to 2.452, *P =* 0.011). In addition, the stronger IgG fluorescence intensity conferred an OR of 3.881 (2.652–5.680, *P <* 0.001). The condition of the female gender and the fluorescence intensity of IgG were similar in group B. The chance of IgA and IgG co-deposition in female cases was higher than that in males (OR = 1.382, 95% CI, 1.053 to 1.814, *P =* 0.002), and patients with stronger IgG deposition were 2.673 times more likely to have IgA and IgG co-deposition compared with patients with less than 3+ IgG deposition (95% CI, 2.018–3.541, *P <* 0.001). Moreover, mucosa tissue increased the OR of IgA and IgG co-deposition by 3.052 (95% CI, 2.315–4.025, *P <* 0.001) compared to skin in group B.

**Table 1:** Factors associated with IgA and IgG co-deposition.

Variable	OR	95% CI	*P*-value
Group A			
Age ≥ 60	1.086	0.712 to 1.657	0.751
Female gender	1.665	1.131 to 2.452	0.011*
Sites involvement			
Skin	Reference		
Mucosa	1.191	0.829 to 1.713	0.355
Severity of IgG deposits^a^			
Weak and normal	Reference		
Strong	3.881	2.652 to 5.680	< 0.001*
Group B			
Age ≥ 60	1.045	0.795 to 1.374	0.780
Female gender	1.382	1.053 to 1.814	0.020*
Sites involvement			
Skin	Reference		
Mucosa	3.052	2.315 to 4.025	< 0.001*
Severity of IgG deposits			
Weak and normal	Reference		
Strong	2.673	2.018 to 3.541	< 0.001*

The association of a series of effective factors was evaluated with logistic regression models. *P-*value <0.05 is considered significant and changed to bold. OR, odds ratio; CI, confidence interval.

The severity of deposits was determined by two pathologists using a semi-quantitative scoring method, ranging from 1+ (weakest) to 4+ (strongest). Weak: 1+, normal: 2+, strong: more than 3+.

*P < 0.05.

Univariable analysis.

### The relationship between the disease severity, tissue involvement, and prognosis of IgA and IgG co-deposition

In group A, compared with IgG deposition only, patients with IgA and IgG co-deposition had a higher proportion of moderate to severe PDAI scores (61.02% vs. 46.60%, *P =* 0.036), but there were no significant differences between the two groups in antibody titers, mucosal involvement, or treatment response. In group B, patients with IgA and IgG co-deposition exhibited significantly higher prevalence of mucosal involvement compared to those with IgG deposition alone (54.35% vs. 32.43%, *P =* 0.007). Although this cohort showed a higher rate of mild disease severity as assessed by BPDAI scores (71.74% vs. 62.84%), no significant intergroup difference was observed in overall disease severity (*P =* 0.269). In addition, the proportion of disease severity scores improvement after 6 months of standard treatment did not demonstrate significant intergroup variation. The results of the retrospective clinical characteristics analysis are presented in **Table 2**.

**Table 2:** Clinical characteristics of IgA and IgG co-deposition and IgG deposition only.

Variable	IgA and IgG co-deposition	IgG deposition only	*P*-value
Group A			
Disease severity (PDAI Score)			
Mild, *n* (%)	23 (38.98%)	259 (53.40%)	0.036*
Moderate to Severe, *n* (%)	36 (61.02%)	226 (46.60%)	
Disease severity (Pemphigus-specific antibody titers)			
Dsg1, Median (IQR), u/ml	90.10 (56.70–143.73)	101.59 (32.07–167.01)	0.998
Dsg3, Median (IQR), u/ml	118.04 (16.27–167.41)	132.41 (57.28–168.22)	0.261
Mucosal involvement			
No mucosal involvement, *n* (%)	22 (43.14)	161 (39.75)	0.642
Mucosal involvement, *n* (%)	29 (56.86)	244 (60.25)	
Change of disease severity (PDAI score)			
Improvement, *n* (%)	31 (79.49%)	221 (82.77%)	0.615
No improvement or worsening, *n* (%)	8 (20.51%)	46 (17.23%)	
Group B			
Disease severity (BPDAI score)			
Mild, *n* (%)	33 (71.74%)	93 (62.84%)	0.269
Moderate to severe, *n* (%)	13 (28.26%)	55 (37.16%)	
Mucosal involvement			
No mucosal involvement, *n* (%)	21 (45.65)	100 (67.57)	0.007*
Mucosal involvement, *n* (%)	25 (54.35)	48 (32.43)	
Change of disease severity (BPDAI score)			
Improvement, *n* (%)	11 (64.71%)	48 (75.00%)	0.540
No improvement or worsening, *n* (%)	6 (35.29%)	16 (25.00%)	

Continuous variables are performed as Mean (SD) for normally distributed data and as median (IQR) for non-normally distributed data. Categorical variables as count (%). Accurate statistics are chosen for the data, including one-way analysis of variance (ANOVA), and Pearson’s χ2 test (or Fisher’s exact test). *P*-value < 0.05 is considered significant and changed to bold.

PDAI, Pemphigus Disease Area Index; BPDAI, Bullous Pemphigoid Disease Area Index; Dsg, desmoglein.

*P < 0.05.

## Discussion

In this study, we analyzed the immunofluorescence patterns and demographic characteristics of patients in AIBD-WCH, as well as the associations between disease severity, prognosis, and IgA and IgG co-deposition. Compared to cases with IgG deposition only, our study found that in cases of linear deposition, the combination of IgG and IgA deposition was associated with a higher percentage of female patients, mucosal involvement, and stronger IgG intensity. Similar outcomes were observed in net-like deposition patterns, and IgA and IgG co-deposition was associated with higher PDAI scores, despite no significant differences in site involvement.

Basically, the deposition of IgA alone is considered indicative of IgA pemphigus or LABD, whose clinical features are distinct from those of pemphigus or pemphigoid (9, 10). However, if there is IgG deposition at the same time, the diagnosis is usually pemphigus or pemphigoid. In terms of the pathogenesis of pemphigus and pemphigoid, IgG is generally considered a pathogenic antibody (16), whereas the role of IgA in pathogenesis is less frequently discussed.

Some reports named IgA and IgG co-deposition in BMZ as linear IgA/IgG bullous dermatitis, emphasizing the pathogenic role of IgA (11). There are also reports describing IgA/IgG pemphigus as a variant of IgG pemphigus rather than IgA pemphigus (12, 13). Although no prior research has statistically determined the frequency of IgA co-deposits among IgG-positive DIF results, our findings indicate that such co-deposition is relatively common.

Interestingly, we found that the proportion of women with IgG and IgA co-deposition was higher than that with IgG deposition only, while no gender preference was reported in the cases of IgG/IgA pemphigus or linear IgA/IgG bullous dermatitis (17). However, in our previous study, females accounted for 55% (*n* = 496) of the pemphigus cohort (18). The difference of gender in AIBD requires further investigation.

Co-deposition of IgA and IgG in mucosal specimens was more frequent in our study, which may be related to the secretion of IgA by mucosal cells. As the secretory form, SIgA is secreted by mucosal cells without passing through the intercellular spaces between mucosal epithelial cells (19, 20), meaning these antibodies are rarely detected intercellularly. This may explain our observation that IgA co-deposition in the BMZ rather than intercellular was associated with mucosa tissue.

In our study, IgG/IgA co-deposition correlated with stronger IgG staining intensity—a feature previously linked to disease activity (21). Clinical analysis in group A revealed higher PDAI scores in the co-deposition subgroup, despite antibody titer results showing no significant differences. Supporting this, certain investigations have demonstrated elevated epidermal expression of IL-8 and MMP-9 in IgG/IgA pemphigus patients compared to traditional pemphigus cases (22), potentially explaining the more severe clinical manifestations associated with IgG/IgA co-deposition.

In group B, IgG/IgA co-deposition was also associated with increased IgG staining intensity, consistent with earlier studies that associated IgA presence with more severe clinical profiles in mucous membrane pemphigoid (23, 24). However, we observed a trend toward higher proportions of mild cases in the co-deposition group, though this finding did not reach statistical significance. In some pemphigoid subtypes, IgG and/or IgE are known to trigger complement- and Fcγ receptor-mediated inflammatory pathways as key pathogenic mechanisms (25). In contrast, IgA is generally considered inefficient at complement activation due to the absence of C1q-binding residues in its Fc region (26). Notably, IgA exhibits dual immunomodulatory roles, capable of inducing both pro-inflammatory and immunosuppressive responses (27). For instance, monomeric IgA binding to FcαRI has been shown to inhibit IgG-mediated phagocytosis, chemotaxis, bactericidal activity, oxidative burst, and cytokine release (28). In addition, another potential explanation lies in our focus on broad fluorescence patterns rather than detailed pemphigoid subgroup analyses, which may have obscured subtype-specific correlations between IgA and disease severity.

We also collected data on the changes in disease severity scores after 6 months of standard treatment in different subgroups. However, there was no significant difference in the proportion of score improvement between groups. Whether co-deposition of IgA and IgG affects treatment response requires further study.

Although this is a relatively large-sized study, the method of retrospective analytical cross-sectional study has its limitations. Moreover, we did not control some confounding factors, such as medications or genetic predisposition. This needs to be improved in future studies. Although we made conjectures based on these results, further exploration of the mechanisms through experiments is necessary.

## Conclusion

In conclusion, our study found that IgG and IgA co-deposition was a common pattern in these cases. Compared with IgG deposition only, female gender, stronger IgG deposition, and mucosa tissue are key factors affecting IgA co-deposition in AIBD patients. IgA and IgG co-deposition is associated with the severity of pemphigus and mucosal involvement in pemphigoid. These findings suggest that IgA may play an underappreciated role in the pathogenesis of these diseases.

## Data Availability

The original contributions presented in the study are included in the article/**Supplementary Material**. Further inquiries can be directed to the corresponding authors.

## Supplementary material

Supplementary Table 1 The demographic characteristics and immunofluorescence patterns of AIBD patients. Continuous variables are performed as Mean (SD) for normally distributed data and as median (IQR) for non-normally distributed data. And categorical variables as count (%). Accurate statistics are chosen for the data, including one-way analysis of variance (ANOVA), and Pearson’s χ2 test (or Fisher’s exact test). *P*-value <0.05 is considered significant and changed to bold. a. Others refer to other deposition patterns: IgM, C3, IgM + IgG, IgA + IgM. b. The fluorescence intensity was evaluated with an objective assessment of the pathologist scored from 1+ (weakest) to 4+ (strongest). Weak: 1+, normal: 2+, strong: more than 3+. NA not available. **P* < 0.05.
