# Supplementary material for: Demographic characteristics and disease severity associated with IgA/IgG deposition patterns in autoimmune bullous diseases: a cohort study based on a registry database
Source: Front Immunol. 2025 May 26;16:1565073. doi: 10.3389/fimmu.2025.1565073 (PMC12146370; doi:10.3389/fimmu.2025.1565073)
Supplement: Supplementary Table 1 — The demographic characteristics and immunofluorescence patterns of AIBD patients. Continuous variables are performed as Mean (SD) for normally distributed data and as median (IQR) for non-normally distributed data. And categorical variables as count (%). Accurate statistics are chosen for the data, including one-way analysis of variance (ANOVA), and Pearson’s χ2 test (or Fisher’s exact test). P-value <0.05 is considered significant and changed to bold. a. Others refer to other deposition patterns: IgM, C3, IgM + IgG, IgA + IgM. b. The fluorescence intensity was evaluated with an objective assessment of the pathologist scored from 1+ (weakest) to 4+ (strongest). Weak: 1+, normal: 2+, strong: more than 3+. NA not available. *P < 0.05. [file Table1.docx]

# Supplementary Table 1. The demographic characteristics and immunofluorescence patterns of AIBD patients

|  | Total | IgA | IgG | IgA + IgG | IgA + IgG + IgM | Others ^a^ | *P*-value |
| --- | --- | --- | --- | --- | --- | --- | --- |
| **Group A** | | | | | | | |
| N | 1512 | 9 | 1310 | 128 | 9 | 56 | / |
| Female gender, n (%) | 874 (57.80) | 8 (88.89) | 734 (56.03) | 87 (67.97) | 8 (88.89) | 37 (66.07) | **0.003** |
| Age, *Median (IQR)*, years | 50.00 (41-60) | 52.00 (31.50-58) | 50.00 (41-60) | 51.00 (43-59) | 48.00 (30-61) | 47.00(38-54.50) | 0.270 |
| Mucosa, n (%) | 720 (47.62) | 0 (0.00) | 608 (46.41) | 65 (50.78) | 3 (33.33) | 44 (78.57) | **<0.001*** |
| Fluorescence intensity of IgG ^b^ | | | | | | | |
| Weak, n (%) | 37 (2.48) | NA | 31 (2.37) | 3 (2.34) | 0 (0.00) | 2 (4.26) | **<0.001*** |
| Normal, n (%) | 931 (62.36) | NA | 857 (65.42) | 42 (32.81) | 1 (11.11) | 31 (65.96) |  |
| Strong, n (%) | 525 (35.16) | NA | 422 (32.21) | 83 (64.84) | 8 (88.89) | 12 (25.53) |  |
| **Group B** | | | | | | | |
| N | 1738 | 50 | 792 | 295 | 96 | 505 | / |
| Female gender, n (%) | 974 (56.04) | 31 (62.00) | 415 (52.40) | 178 (60.34) | 60 (62.50) | 290 (57.43) | 0.056 |
| Age, *Median (IQR)*, years | 59.00 (46-70) | 53.00 (45-65) | 65.00 (53-74) | 63.00 (53-70) | 42.50 (36-70 ) | 47.00 (30-61) | **<0.001*** |
| Mucosa, n (%) | 654 (37.63) | 17 (34.00) | 271 (34.22) | 181 (61.36) | 40 (41.67) | 145 (28.71) | **<0.001*** |
| Fluorescence intensity of IgG | | | | | | | |
| Weak, n (%) | 106 (6.10) | NA | 84 (10.61) | 9 (3.05) | 2 (2.08) | 11 (12.09) | **<0.001*** |
| Normal, n (%) | 684 (42.35) | NA | 500 (63.13) | 147 (49.83) | 37 (38.54) | 52 (57.14) |  |
| Strong, n (%) | 432 (24.86) | NA | 208 (26.26) | 139 (47.12) | 57 (59.38) | 28 (30.77) |  |

Continuous variables are performed as Mean (SD) for normally distributed data and as median (*IQR*) for non-normally distributed data. And categorical variables as count (%). Accurate statistics are chosen for the data, including One-way analysis of variance (ANOVA), and Pearson’s χ2 test (or Fisher's exact test). *P*-value< 0.05 is considered significant and changed to bold.

a. Others refer to other deposition patterns: IgM, C3, IgM + IgG, IgA + IgM.

b. The fluorescence intensity was evaluated with an objective assessment of the pathologist scored from 1+ (weakest) to 4+ (strongest). Weak: 1+, normal: 2+, strong: more than 3+.

Abbreviations: NA not available.

**P*< 0.05
